# Association between musculoskeletal function deterioration and locomotive syndrome in the general elderly population: a Japanese cohort survey randomly sampled from a basic resident registry

**DOI:** 10.1186/s12891-020-03469-x

**Published:** 2020-07-03

**Authors:** Ryosuke Tokida, Shota Ikegami, Jun Takahashi, Yoshikazu Ido, Ayaka Sato, Noriko Sakai, Hiroshi Horiuchi, Hiroyuki Kato

**Affiliations:** 1 Rehabilitation Center, Shinshu University Hospital, Asahi 3-1-1, Matsumoto, Nagano, 390-8621 Japan; 2 Department of Orthopaedic Surgery, Shinshu University School of Medicine, Asahi 3-1-1, Matsumoto, Nagano, 390-8621 Japan; 3 Department of Orthopaedic Surgery, New Life Hospital, Obuse, Kamitakai-gun, Nagano, 381-0295 Japan

**Keywords:** Epidemiological study, Cross-sectional survey, Prevalence, Sarcopenia, Locomotion, physical examination

## Abstract

**Background:**

Locomotive syndrome (LoS) is defined as the loss of mobility due to age-related impairment of motor organs. The purpose of this study was to evaluate the prevalence and severity of LoS, muscular strength and balancing ability, and prevalence of sarcopenia in relation to the presence of LoS according to sex and age groups ranging between 50 and 89 years.

**Methods:**

Male and female participants between the ages of 50–89 were randomly selected in the resident registry of a cooperating town. Calls for participation continued until approximately 50 consenting participants were successfully recruited for each age group and sex. A total of 413 participants (203 male and 210 female) were enrolled for undergoing a LoS risk test and measuring their physical function. Physical function was compared to participants with or without LoS.

**Results:**

A total of 312 patients (75.5%) were diagnosed as LoS, of which 144 (46.2%) were male and 168 (53.8%) were female. The severity of LoS for the 312 patients were 210 (67.3%) for stage 1 and 102 (32.7%) for stage 2. The prevalence of LoS in males were 37, 59, 91, and 100% in the 50s, 60s, 70s, and 80s age strata, respectively. The prevalence of LoS in females were 71, 62, 89, and 98% in the 50s, 60s, 70s, and 80s age strata, respectively. The prevalence of sarcopenia was significantly higher as the age strata in males grew higher. Knee extension strength was significantly lower for participants in their 50s and females in addition to females in their 60s with LoS. The 31 patients diagnosed as sarcopenia included 29 (93.5%) with LoS, 11 (35.4%) classified as LoS stage 1, and 18 (58.1%) classified as stage 2.

**Conclusions:**

The prevalence of LoS was high in participants over 70 years of age. In males, the prevalence of sarcopenia was higher as the age strata grew higher. Patients with LoS exhibited lower knee extension strength. We believe that some measures to prevent or improve LoS may require exercise to increase the muscle strength of the lower limbs.

## Background

Japan is one of the most aged countries in the world, and the growth of its elderly population that require nursing care has become a significant social and financial problem. In 2007, the Japanese Orthopaedic Association proposed the term “locomotive syndrome” (LoS) to describe the loss of mobility due to age-related impairment of motor organs, and the organization subsequently launched an early detection and preventative care program [1, 2]. Preventative care for LoS is important, as there is an increased risk of requiring nursing care with the progression of LoS.

In the past, only subjective evaluations such as a “loco-check” (a neologism combining “locomotive syndrome” and “check”) and the 25-question geriatric locomotive function scale (25-qGLFS) [3] were conventionally used as a method for detecting LoS. However, the LoS risk test was developed in 2013 to enable objective evaluations, comprising of three risk assessments that include the stand-up test (SUT), two-step test (TST), and 25-qGLFS. The severity of LoS can be classified into two stages under the LoS risk test, wherein LoS Stage 1 represents a mild stage that exhibits the initial loss of mobility and LoS Stage 2 represents a severe stage that exhibits a progressive loss of mobility [4]. Although surveys on the prevalence and severity of LoS in middle-aged and elderly patients using the LoS risk test have been reported in the literature [5–8], to the best of our knowledge, only one report conducted examinations according to each age group and sex [5]. In addition, despite reports that suggest the muscle strength of the lower limbs, balancing ability, and walking ability were significantly related to LoS [9–11], few studies have examined the muscular strength and balancing ability in relation to the presence of LoS according to each age group and sex [5]. The first aim of this study was to clarify the association between physical function and LoS.

Sarcopenia is a condition characterized by muscle weakness, decline of motor ability, or loss of muscle mass due to aging [12]. In addition, sarcopenia is reported to be common in patients requiring long-term care and patients with motor disorders [13]. The clinical features of LoS and sarcopenia were closely linked to each other [14]. However, reports on the relationship between LoS and sarcopenia are scarce in the literature [15]. The second aim of this study was to clarify the association between LoS and sarcopenia.

Taking account of the background described above, we performed a cross-sectional study on the musculoskeletal function of a town resident. We evaluated the prevalence and severity of LoS according to sex and age groups ranging from 50 to 89 years, as well as the prevalence of sarcopenia. Furthermore, we assessed the knee extension strength, balancing ability, and sarcopenia in relation to LoS.

## Methods

### Subjects

**Fig. 1:**
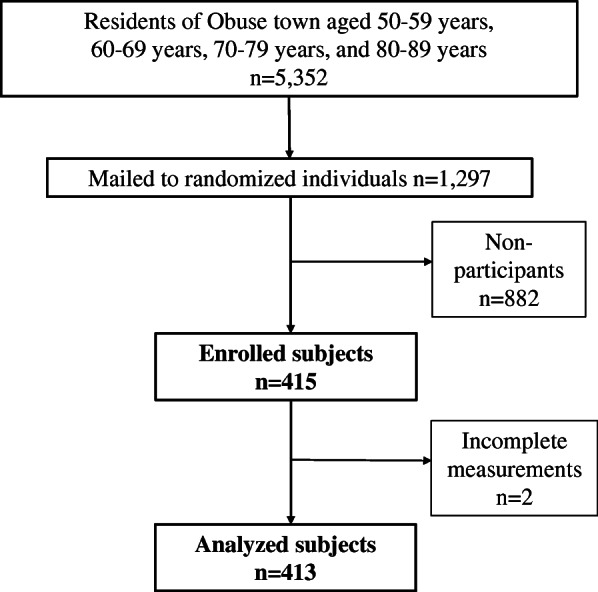
Flow-chart of participants from a cooperating town

This was cross-sectional study for observational research. From October 2014 to June 2017, we conducted an epidemiological study of residents (the Obuse Study) as a joint collaboration with a cooperating town office. Male and female participants between the ages of 50–89 were randomly selected from a pool of 5352 registrants in the resident registry of the cooperating town, which locates in a rural area. Those selected from the registry were asked whether they would be able to undergo a physical examination, and calls for participation were continued until approximately 50 consenting participants were successfully recruited for each age group, which were 50’s, 60’s, 70’s, and 80’s, and sex. Four hundred and fifteen participants were enrolled in the Obuse study. Four hundred and thirteen participants were consequently included in the study, excluding 2 participants with incomplete measurements (Fig. 1). Prior to this study, we had not performed a calculation to establish a statistical sample size based on multiple outcome indices. A feasible survey was conducted based on the balance between financial costs and time, number of staff, and participants.

### Assessing the presence / absence of LoS and its severity

Prior to the physical examination, we mailed and requested a 25-qGLFS questionnaire [3] to be filled out by our participants. These self-reported questionnaires were collected on-site, where the examination was held. The SUT [16] and TST [17] were conducted during the examination. The SUT evaluates lower-body muscle strength by measuring whether the subject can rise from a sitting position on boxes of heights of 40, 30, 20, and 10 cm with 1 or both legs. The relative difficulty of standing from each height, from least to most challenging, was as follows: 40 cm, both legs standing (level 1); 30 cm, both legs standing (level 2); 20 cm, both legs standing (level 3); 10 cm, both legs standing (level 4); 40 cm, single-leg standing (level 5); 30 cm, single-leg standing (level 6); 20 cm, single-leg standing (level 7); and 10 cm, single-leg standing (level 8). The successfully completed task with the greatest difficulty level was regarded as the subject’s final grade. The 2-step test evaluated the total length of 2 maximally long strides (in centimetres) divided by body height (in centimetres).

LoS were established in two stages. From these three tests, the participants were classified into no LoS, LoS Stage 1, or LoS Stage 2 group. LoS stage 1 was defined as a TST score < 1.3, difficulty with one-leg standing (OLS) from a 40 cm seat in the SUT (either leg), or a 25-qGLFS score ≥ 7. LoS stage 2 was defined as a TST score < 1.1, difficulty with standing from a 20 cm seat using both legs in the SUT, or a 25-qGLFS score ≥ 16. LoS stage 1 was diagnosed as starting to decline in mobility, and LoS stage 2 was diagnosed as progressing to a decline in mobility [4].

### Evaluation of physical function

The muscle strength for the lower limb was measured by a performance recorder (HUR ®, Kokkola, Finland) and leg extension/curl Rehab 5530 (HUR ®, Kokkola, Finland). The validity and reliability of these instruments have been reported [18] and have been commonly used in previous assessments of muscle strength [19, 20]. The right and left leg extension power (kg) was divided by weight (kg), and their mean value was defined as the knee extension strength. Balancing ability was measured by measuring the total duration the subject could stay in a balancing position while keeping his or her eyes open and standing on either leg [21]. At a maximum of 60 s, the left and right mean values were defined as the OLS test (sec). The grip strength was measured using a grip strength meter (Jamar Hand Dynamometer, Sammons Preston Rolyan^@^, Bolingbrook, Ill, USA), and the mean value on the left and right were defined as the grip strength.

### Assessment of sarcopenia

We assessed the muscle mass and strength based on the criteria proposed by the Asian Working Group for Sarcopenia [12]. For determining the muscle mass, we used a Dual-Energy X-Ray Absorptiometry (GE Prodigy, GE healthcare, Chicago, IL, USA) to measure and evaluate the skeletal muscle mass index (SMI). In case the walking speed was less than 0.8 m/s or the grip strength was less than 26 kg in males and less than 18 kg in females, the muscle mass was measured. The cut-off for muscle mass was 7.0 kg/m^2^ in males and 5.4 kg/m^2^ in females. Sarcopenia can be diagnosed based on having a low muscle mass that falls below the cut-off value. In the present study, we could not measure the walking speed due to timewise and spatial restrictions, so we used the grip strength as a primary physical performance assessment.

### Statistical analysis

The prevalence of LoS for each age group and sex was compared to the prevalence of sarcopenia using the Steel-Dwass test. Knee extension strength and OLS test were compared to participants with or without LoS using the Student’s t-test. The relationship between LoS and sarcopenia was analysed using the Fisher’s exact test. Sensitivity, specificity, and positive / negative likelihood ratio of LoS diagnosis by sarcopenia were calculated. A positive likelihood ratio of more than 5 and a negative likelihood ratio of less than 0.2 were considered useful for diagnosis. All statistical analyses were performed with EZR (Saitama Medical Center, Jichi Medical University) [22], which is a graphical user interface for R (version 3.5.2; The R Foundation for Statistical Computing, Vienna, Austria). *P* < 0.05 was considered to be statistically significant.

## Results

The baseline characteristics and functions of 413 participants are shown for each age group and sex in Table 1. The number of male participants were 50 for those aged 50–59 years, 53 for 60–69 years, 55 for 70–79 years, and 45 for 80–89 years. The number of female participants were 47 for those aged 50–59 years, 61 for 60–69 years, 54 for 70–79 years, and 48 for 80–89 years. There were many tertiary industry workers up to retirement at age 65 and agricultural workers in the post-retirement age group. The older people were, the more likely they were classified to be unemployed.

**Table 1:** Characteristics of 413 subjects in the Obuse study cohort

	Age strata (years)	N	Height (cm)	Weight (kg)	BMI (kg/m^2^)	SMI (kg/m^2^)	Job (Pri;Sec;Ter;None)
Male	50–59	50	171.8 (5.9)	67.1 (9.0)	22.7 (2.9)	7.6 (0.7)	3; 7; 40; 0
	60–69	53	166.7 (4.7)	66.9 (7.7)	24.1 (2.7)	7.5 (0.6)	18; 5; 19; 11
	70–79	55	163.2 (4.9)	60.0 (10.2)	22.5 (3.4)	7.0 (0.7)	22; 2; 8; 23
	80–89	45	160.1 (5.6)	57.5 (8.4)	22.4 (2.7)	7.0 (0.9)	19; 0; 3; 23
	Total	203	165.6 (6.8)	63.0 (9.8)	22.9 (3.0)	7.3 (0.8)	62; 14; 70; 57
Female	50–59	47	158.1 (4.9)	55.4 (8.9)	22.2 (3.8)	5.9 (0.6)	5; 4; 29; 9
	60–69	61	152.8 (5.3)	52.2 (7.6)	22.3 (2.7)	5.9 (0.5)	21; 4; 17; 19
	70–79	54	149.7 (5.3)	50.6 (7.9)	22.6 (3.2)	6.1 (0.6)	16; 4; 8; 26
	80–89	48	144.6 (5.8)	48.3 (7.8)	23.1 (3.3)	6.1 (0.7)	11; 0; 5; 32
	Total	210	151.3 (7.1)	51.6 (8.4)	22.5 (3.2)	6.0 (0.6)	53; 12; 59; 86

Note: Values represent mean (standard deviation).

Abbreviations: *BMI* Body Mass Index; *SMI* Skeletal Muscle Index; *Pri* Primary industry employment; *Sec* Secondary industry employment; *Ter* Tertiary industry employment

The physical functions of participants are shown for each age group and sex in Table 2. All physical performance tests revealed inferior results on average for higher age groups. These physical characteristics of each group divided by age and sex demonstrated approximately the same value in 2014 for the Japanese population [23]. Three hundred and twelve patients (75.5%) were diagnosed as LoS, of which 144 (46.2%) were male and 168 (53.8%) were female. The prevalence of LoS was 53.6% for patients aged 50–59 years, 60.5% for 60–69 years, 90.8% for 70–79 years, and 98.9% for 80–89 years. The severity of LoS for the 312 patients were 210 (67.3%) for LoS Stage 1 and 102 (32.7%) for LoS Stage 2. More than half of the participants aged 50–59, 60–69, and 70–79 were LoS Stage 1. In both males and females, there was a significantly higher prevalence of LoS in older age groups, e.g., those over 60 years of age.

**Table 2:** Results of physical performance tests in each subject group

	Age strata (years)	Knee extension strength	OLS time (sec)	Grip strength (kg)	SUT	TST score	25-qGLFS score
Male	50–59	1.8 (0.5)	51.5 (13.4)	40.0 (6.9)	5	1.6 (0.2)	2.9 (3.5)
	60–69	1.7 (0.5)	46.0 (16.9)	36.8 (6.0)	4	1.5 (0.2)	5.1 (5.6)
	70–79	1.3 (0.4)	24.6 (17.1)	31.1 (5.2)	4	1.4 (0.2)	9.4 (11.1)
	80–89	1.0 (0.4)	13.2 (13.7)	25.1 (5.2)	3	1.1 (0.3)	14.3 (14.1)
Female	50–59	1.4 (0.4)	46.9 (16.2)	25.4 (5.2)	4	1.5 (0.1)	5.0 (4.8)
	60–69	1.2 (0.4)	43.2 (17.0)	21.8 (3.9)	4	1.4 (0.1)	4.4 (4.6)
	70–79	0.9 (0.3)	23.0 (15.9)	20.5 (3.9)	3	1.3 (0.4)	13.9 (15.8)
	80–89	0.8 (0.3)	10.9 (12.4)	17.1 (3.6)	3	1.1 (0.3)	18.9 (14.8)

Note: Values represent mean (standard deviation).

Abbreviations: *OLS* One-leg standing; *SUT* Stand-up test; *TST* Two-step test; *25-qGLFS* 25-Question geriatric locomotive function scale

**Fig. 2:**
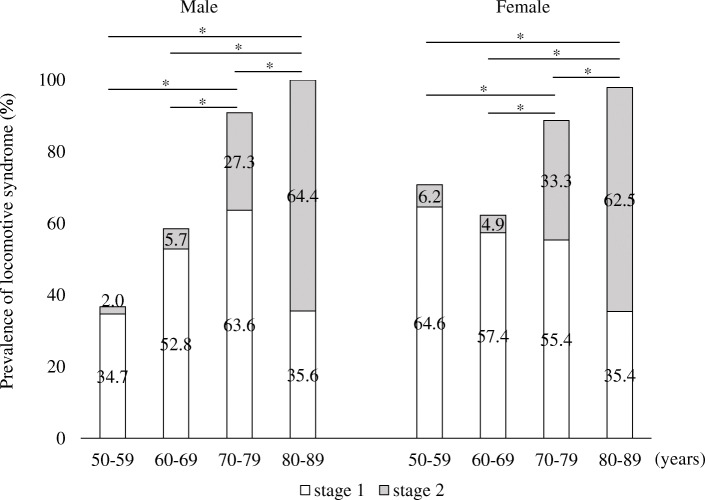
Prevalence of locomotive syndrome. *p < 0.05

Over 60% of both male and female participants aged 80–89 years were LoS Stage 2. The prevalence of LoS and LoS Stage 2 significantly increased with age (Fig. 2). Due to difficulties in maintaining the one-leg stand from a 40-cm-high seat during the SUT, many participants were diagnosed as LoS under the LoS risk test (Table 3).

**Table 3:** Prevalence by age and sex as evaluated in the locomotive syndrome risk test

	Age strata (years)	N	Unable to complete one-leg SUT from ≥40 cm	TST < 1.3	25-qGLFS score ≥ 7
Male	50–59	50	14 (28.0%)	2 (4.0%)	6 (12.0%)
	60–69	53	28 (52.8%)	5 (9.4%)	12 (22.6%)
	70–79	55	49 (89.1%)	16 (29.1%)	22 (40.0%)
	80–89	45	45 (100%)	32 (71.1%)	28 (62.2%)
Female	50–59	47	26 (55.3%)	1 (2.1%)	13 (27.7%)
	60–69	61	32 (52.5%)	8 (13.1%)	12 (19.7%)
	70–79	54	48 (88.9%)	20 (37.0%)	30 (55.6%)
	80–89	48	46 (95.8%)	37 (77.1%)	41 (85.4%)

Note: Values represent number (prevalence)

Abbreviations: *SUT* Stand-up test; *TST* Two-step test; *25-qGLFS* 25-Question geriatric locomotive function scale

**Fig. 3:**
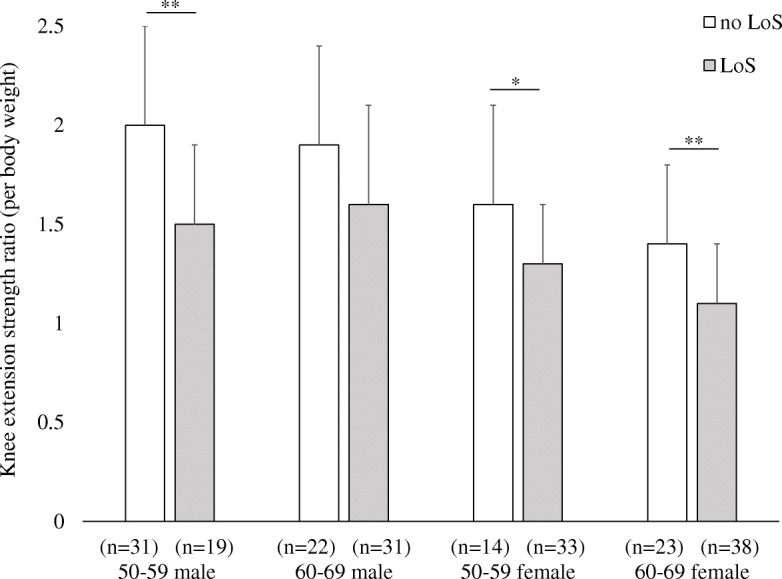
Comparison of knee extension strength between participants indicated no-locomotive syndrome and those indicated locomotive syndrome. Values represent mean (standard deviation). LoS, locomotive syndrome. *p < 0.05; **p < 0.01

**Fig. 4:**
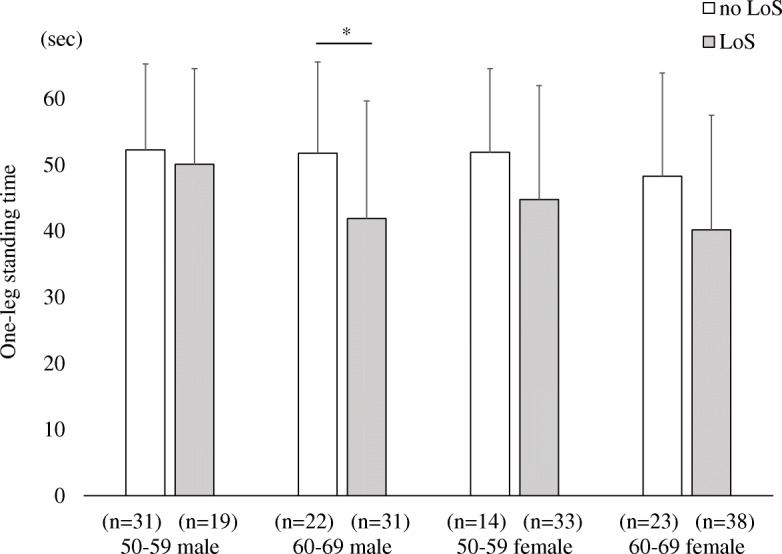
Comparison of one-leg standing test between participants indicated no-locomotive syndrome and those indicated locomotive syndrome. Values represent mean (standard deviation). LoS, locomotive syndrome. *p < 0.05

In comparing the knee extension strength and OLS test between the no LoS and LoS groups, the knee extension strength was significantly lower in the LoS group in males and females aged 50–59 years and females aged 60–69 years (Fig. 3). OLS test was significantly lower in value for males aged 60–69 in the LoS group (Fig. 4). We were unable to compare participants aged 70–79 and 80–89 because participants were mostly classified under the LoS group.

**Fig. 5:**
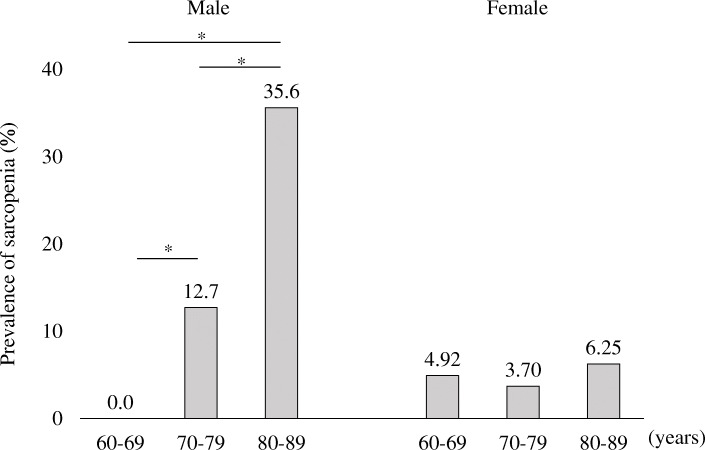
Prevalence of sarcopenia. *p < 0.05

The total number of participants diagnosed as sarcopenia was 31 (9.81%), 23 of which were male (11.3%) and 8 of which were female (4.91%). The prevalence of sarcopenia also increased with age in males, consisting of 0% for participants aged 60–69 years, 12.7% for participants aged 70–79, and 35.6% for participants aged 80–89. In male participants, the prevalence of sarcopenia was significantly higher as the age strata grew higher (Fig. 5).

Forty-nine patients diagnosed with sarcopenia included 29 (93.5%) with LoS, 11 (35.5%) classified as LoS stage 1, and 18 (58.1%) classified as stage 2. Participants with sarcopenia tended to have LoS, but the association was not significant (odds ratio [OR] 3.4, 95% confidence interval [95% CI] 0.8 to 14.6, *P* = 0.10). The OR for males and females were 5.5 (95%CI 0.7 to 42.7, *P* = 0.10) and 1.5 (95%CI 0.2 to 13.1, *P* = 0.69), respectively. There were no significant associations for both sex. Table 4 shows whether the presence of sarcopenia can predict the presence of LoS. Although the screening for LoS via diagnosis of sarcopenia was insensitive and often overlooked, the specificity was higher at 95% or more. The positive/negative likelihood ratio were 3.12/0.92, respectively, which were insufficient for predicting LoS based on the diagnosis of sarcopenia.

**Table 4:** Screening for locomotive syndrome based on the diagnosis of sarcopenia

	Sensitivity	Specificity	Positive likelihood ratio	Negative likelihood ratio
Male	17% (15–18%)	96% (83–99%)	4.71 (0.91–27.4)	0.86 (0.82–1.01)
Female	5% (3–6%)	97% (87–99%)	1.51 (0.26–9.39)	0.98 (0.95–1.11)
Total	11% (10–12%)	96% (89–99%)	3.12 (0.88–11.8)	0.92 (0.89–1.01)

Note: Values represent with (95% confidence intervals)

## Discussion

In the present study, we evaluated the prevalence of LoS and sarcopenia. Furthermore, we assessed the knee extension strength, balancing ability, and sarcopenia in relation to LoS. The total number of participants was 312 (75.5%) for those diagnosed as LoS and 31 for those diagnosed as sarcopenia (9.81%). Knee extension strength and OLS test between the no LoS and LoS groups, knee extension strength was significantly lower in the LoS group. Particularly in males, sarcopenia patients could be assumed to have LoS, but non-sarcopenia patients may or may not have LoS.

A characteristic feature of this study was the collection of participants with age ranging from 50 to 89 years by randomly sampling from the resident register. Compared to conventional research on residents that recruits active volunteers, this research was designed to create a cohort that more accurately reflects common residents. In addition, another unique feature of this study was that we gathered approximately 50 participants for physical examinations in each age group and sex, and as a result, the age and male-female ratio of participants aged 50–89 years were uniformly distributed. This uniform distribution is advantageous for performing accurate statistical comparisons between sexes or age groups.

The prevalence of LoS in this study increased with age, with approximately half of the participants aged 50–59 years and over 90% aged 70–79 years and 80–89 years who were diagnosed as LoS. There were approximately 43,376,300 patients with LoS (75.5% of 50–89 years), 29,195,593 patients (50.8%) with LoS Stage 1, and 14,180,716 patients (24.7%) with LoS Stage 2 in Japan when weighted by the Japanese population. In previous studies [5–8] and in this study, there are differences in the composition ratio for age and sampling method of subjects; thus, cannot simply compare the rate of prevalence for LoS in this study with results from previous studies. However, the prevalence of LoS in this study was comparable to the prevalence of LoS described in a study by Yoshimura et al. [5] that calculated the rate of prevalence by age and sex. On the other hand, when comparing the ratio of participants who were diagnosed with LoS by each criterion in the LoS risk test, the ratio in this study was greater than in the study by Yoshimura et al. in terms of those with difficulties in maintaining a one-leg stand from a 40-cm-high seat during the SUT. In contrast, the ratio of participants who obtained a TST score > 1.3 was less than what was reported by Yoshimura et al. There was no significant difference between both studies in terms of the results in 25-qGLFS. The reason for the difference in results for each criterion in diagnosing LoS is unknown. However, there are reports that suggest that the average step count increases with size of the city [24] and that those who exercise at a moderate or higher intensity are greater in urban areas than in rural areas [25]. We suspect that the results of the SUT in this study were inferior to other studies due to decrease in the muscle strength of knee extension as a result of the comparatively small amount of daily physical activity in residents of rural areas.

When comparing the physical function between the no LoS and LoS groups, the knee extension strength was significantly lower in both male and female participants with LoS aged 50–59 years, and female participants aged 60–69 years. From this fact, we believe that some measures to prevent or improve LoS may require exercise to increase the muscle strength of the lower limbs.

In terms of the prevalence of sarcopenia in the general elderly population of Japan, one report described that the prevalence of sarcopenia according to age group stratifications of 60–64, 65–69, 70–74, 75–79, and ≥ 80 years were 0.5, 0.0, 4.3, 11.2, and 27.0%, respectively [26]. The prevalence of sarcopenia was higher when comparing each age group with past reports. In the present study, the total prevalence of sarcopenia was 9.81%, which was slightly higher than in previous studies. There were approximately 4,031,275 patients with sarcopenia (7.02% of 60–89 years) in Japan when weighted by the Japanese population.

While there have been reports that describe no sex difference in the prevalence of LoS and sarcopenia [6, 26, 27], there have also been reports that describe a higher prevalence of sarcopenia in males compared to females [28]. Significant associations between sarcopenia and LoS were found in both sex based on the results of the ROAD study, which is a study of Japanese residents aged 60 and over [15]. On the other hand, the results of this study showed that males might have an association between sarcopenia and LoS, while females might not. There are several possibilities as to why this difference may have occurred. First, the sample size may have been insufficient. The results for females could be clarified with a larger sample population. However, other causes should also be considered. The cause of LoS, which is more common in females, might have been implicated. For example, osteoporosis and knee osteoarthritis are common in females [6]. Walking speed, which could not be assessed in our study, may contribute to a more severe diagnosis of sarcopenia for females than for males. Moreover, sex differences of the muscle mass cut-off in the diagnostic criteria for sarcopenia might have been affected.

There are several limitations in this study. First, although the research design reduces the sampling bias by adopting random sampling from the resident register, we may not have been able to control for all potential biases as a result of the low participation rate. Furthermore, this study may not reflect the regional characteristics of urban residents, because it is a sampling of one town. Subjects were randomly sampled from the resident registry of the town, but the decision to participate in this study was left to the sampled subjects. Therefore, we conducted a sensitivity analysis on the association between sarcopenia and LoS, assuming that there was heterogeneity in the participation rate depending on whether or not the participants felt impaired in terms of their mobility. While the overall participation rate (32%) remained constant, we changed the participation rates of each cluster, which were LoS (+) / Sarcopenia (+), LoS (+) / Sarcopenia (−), LoS(−) / Sarcopenia(+), and LoS(−) / Sarcopenia(−), to 20, 30, 40, and 50%. These participation rates resulted in an adjusted odds ratio of 4.1 between sarcopenia and LoS, which was larger than the crude odds ratio of 3.4. Conversely, when we changed the participation rates of the above-mentioned clusters to 50, 40, 30, and 20%, the adjusted odds ratio was 4.1, which was also larger than the crude odds ratio of 3.4. Assuming the impact of heterogeneity in participation rates due to the small sample size, it is suggested that we were likely underestimating the relationship between sarcopenia and LoS. Secondly, the diagnosis of sarcopenia is underestimated because we could not measure walking speed in this survey. This has a severe impact on the results of analysis for association between sarcopenia and LoS. Therefore, we performed a sensitivity analysis for sarcopenia misclassification as an additional analysis, and the results are presented in Table 5. The crude odds ratio for sarcopenia/LoS was 3.4 in this study. Because participants with LoS are expected to have low walking speed, the false-negative rate of sarcopenia for LoS-positive participants is likely to be higher than that of LoS-negative participants. Accordingly, the adjusted odds ratio would be greater. Sarcopenia may prove to be a predictor of LoS with the introduction of an appropriate diagnostic procedure for sarcopenia.

**Table 5:** Sensitivity analysis for misclassifications of sarcopenia

Sensitivity of sarcopenia diagnosis in LoS (+)	Sensitivity of sarcopenia diagnosis in LoS (−)					
	100%	90%	80%	70%	60%	50%
100%	3.4	3.0	2.7	2.3	2.0	1.6
90%	3.8	3.4	3.0	2.6	2.2	1.8
80%	4.4	3.9	3.5	3.0	2.6	2.1
70%	5.1	4.6	4.1	3.5	3.0	2.5
60%	6.2	5.5	4.9	4.3	3.6	3.0
50%	7.8	7.0	6.1	5.3	4.5	3.7

Note: Values represent adjusted odds ratio between sarcopenia and locomotive syndrome

Abbreviations: *LoS* Locomotive syndrome

## Conclusion

A characteristic feature of this study was the collection of participants with age ranging from 50 to 89 years by randomly sampling from the resident register. Therefore, this research was designed to create a cohort that more accurately reflects common residents. The prevalence of LoS in this study increased with age, with approximately half of the participants aged 50–59 years and over 90% aged 70–79 years and 80–89 years who were diagnosed as LoS. Due to difficulties in maintaining the one-leg stand from a 40-cm-high seat during the SUT, many participants were diagnosed as LoS. Moreover, the knee extension strength was significantly lower in both male and female participants with LoS aged 50–59 years, and female participants aged 60–69 years. From this fact, we believe that some measures to prevent or improve LoS may require exercise to increase the muscle strength of the lower limbs.

## Data Availability

The datasets used and analysed during the current study are available from the corresponding author on reasonable request.

## Abbreviations

LoS: Locomotive syndrome
25-qGLFS: 25-Question geriatric locomotive function scale
SUT: Stand-up test
TST: Two-step test
OLS: One-leg standing
SMI: Skeletal muscle mass index
BMI: Body mass index
Pri: Primary industry employment
Sec: Secondary industry employment
Ter: Tertiary industry employment
OR: Odds ratio
95% CI: 95% Confidence interval
